# RNA interference and turnover in plants -a complex partnership

**DOI:** 10.3389/fpls.2025.1608888

**Published:** 2025-07-01

**Authors:** Michal Krzyszton, Joanna Kufel, Monika Zakrzewska-Placzek

**Affiliations:** ^1^ Laboratory of Seeds Molecular Biology, Institute of Biochemistry and Biophysics, Polish Academy of Sciences, Warsaw, Poland; ^2^ University of Warsaw, Faculty of Biology, Institute of Genetics and Biotechnology, Warsaw, Poland

**Keywords:** RNA turnover, RNA inteference, miRNA, siRNA, RNA processing

## Abstract

Plants, often exposed to unfavorable external conditions and pathogen attacks, have developed a remarkably complex network of RNA interference (RNAi) pathways. This allows them to adapt gene expression to environmental cues and protects their genomes from invading nucleic acids. The process involves the production of small RNA molecules (sRNAs), which are crucial for ensuring the specificity of this mechanism and ultimately inhibiting the progression of viral infections or the movement of transposons within the genome. The generation of sRNAs is closely linked and balanced with mRNA turnover, as key stages of mRNA synthesis, such as 5’-capping, mRNA maturation, and transcription termination, affect sRNA generation and RNA silencing. Since there are many reviews available on sRNA biogenesis and function, we focused on summarizing the connections between RNA silencing and turnover, explaining how defective RNA maturation or degradation triggers RNA interference. Importantly, RNAi has gained attention as a promising strategy for developing innovative pest control techniques, leveraging this biological mechanism to protect crops. Nonetheless, how the expression of exogenous small RNAs in plants affects the relationship between small RNA and mRNA turnover, as well as how these RNAs are incorporated into specific RNAi pathways, remains uncertain.

## Introduction

RNA interference (RNAi) is an ancient and highly conserved mechanism that protects genomes from invading nucleic acids. This process involves the production of small RNA (sRNA) molecules that bind to effector proteins to ensure precise targeting (Zhan and Meyers, 2023; Vaucheret and Voinnet, 2024). Such specificity is essential for effectively inhibiting viral infections and preventing the disruptive movement of mobile elements, including transposons, within the genome.

Throughout evolution, sRNA pathways have not only safeguarded genome stability but have also been adapted to play pivotal roles in the regulation of gene expression. They operate at both transcriptional (TGS; transcriptional gene silencing) and post-transcriptional (PTGS; post-transcriptional gene silencing) levels, significantly enhancing the capacity of sRNAs to orchestrate a wide range of biological processes, including the regulation of development and adaptations to environmental cues (Li et al., 2017; Brant and Budak, 2018; Singh et al., 2018; Luo et al., 2024; Xu et al., 2024). Consequently, plants have developed a complex network of overlapping sRNA pathways.

The mechanisms of plant sRNA pathways have been extensively studied in the model organism *Arabidopsis thaliana*, with many detailed reviews available on sRNA biogenesis and function (e. g (Li et al., 2017; Brant and Budak, 2018; Lee and Carroll, 2018; Singh et al., 2018; Zhan and Meyers, 2023; Vaucheret and Voinnet, 2024)). This review describes the intricate interactions between RNAi and mRNA turnover, covering aspects such as the synthesis and removal of the mRNA 5′-cap structure, mRNA transcription termination and processing, quality control, and degradation. Additionally, we discuss various triggers of RNA silencing, including aberrant RNAs, while highlighting the crucial roles that diverse RNAi mechanisms play in plant resilience and adaptability.

## A general overview of the RNAi pathways in plants

**Figure 1:**
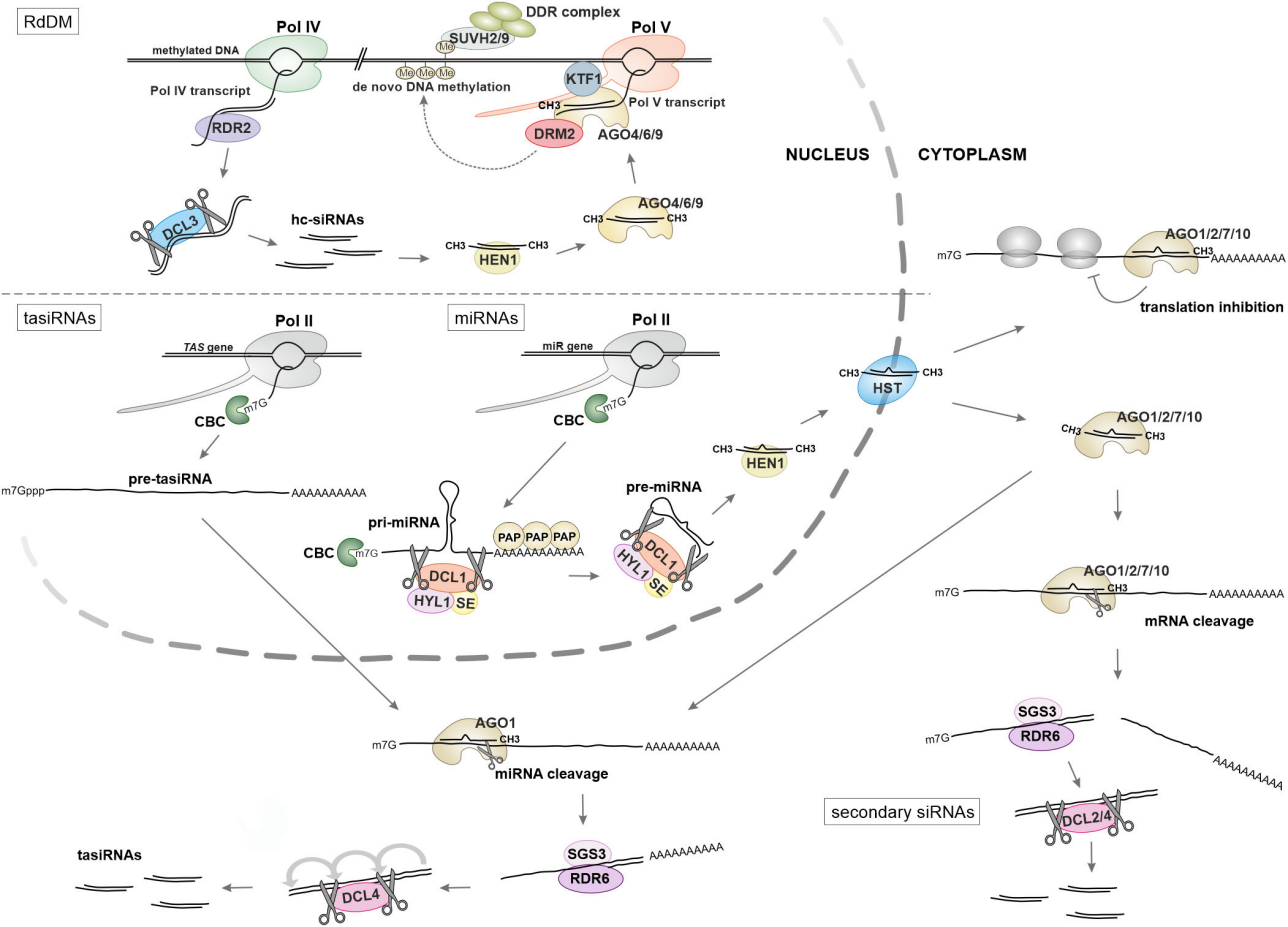
Biogenesis of small RNAs in plants. In the RdDM pathway (upper panel) ncRNA produced by RNA polymerase IV (Pol IV) serves as a substrate for the production of dsRNA by RNA-dependent RNA polymerase RDR6 and subsequent processing into hc-siRNA by DCL3. The transcript synthesized by RNA polymerase V (Pol V) guides a siRNA-loaded silencing complex, which contains an AGO protein, to specific genomic loci. This action initiates DNA methylation and the recruitment of the DDR chromatin-modifying complex. Other classes of sRNAs, miRNAs and tasiRNAs (lower panel) are encoded by their own genes and transcribed by RNA polymerase II (Pol II). Primary pri-miRNA transcripts undergo sequential processing by the microprocessor complex, which consists of three core proteins: HYL1, DCL1, and SE. This processing yields miRNA/miRNA* duplexes, which are then methylated by the HEN1 methyltransferase and transported to the cytoplasm by the exportin HST1. The production of trans-acting siRNAs (tasiRNAs) and secondary siRNAs from dsRNA substrates can be initiated by miRNA-guided cleavage occurring in the cytoplasm. This depends on the slicing activity of AGO proteins, which has been documented for AGO1, AGO2, AGO4, AGO7, and AGO10.

The majority of small RNAs (sRNAs) in Arabidopsis require DICER-LIKE (DCL) endonucleases for their biogenesis from double-stranded RNA (dsRNA) precursors. Additionally, they rely on HEN1 methyltransferase to protect their 3' ends and ARGONAUTE (AGO) proteins to direct sRNA effector complexes to RNAs with complementary sequences (**Figure 1**) (Zhan and Meyers, 2023; Vaucheret and Voinnet, 2024). The source and structure of the dsRNA precursors determine which of the four Arabidopsis DCLs (DCL1-4) most effectively cleaves them into small RNA duplexes of specific lengths: 21 nucleotides (nt) for DCL1 and DCL4, 22 nt for DCL2, and 24 nt for DCL3. This selection process depends on the small RNA duplex's length, structure, and 5' end nucleotide. The resulting double-stranded sRNAs are recruited by one of the ten AGO proteins (AGO1-10), which leads to the selection of guide strands from the RNA duplex (Zhan and Meyers, 2023; Vaucheret and Voinnet, 2024).

Various dsRNA precursors, along with distinct DCL and AGO proteins, coordinate unique pathways driven by several types of sRNAs (**Table 1**). Initially, sRNAs were categorized into two primary groups: microRNAs (miRNAs) and small interfering RNAs (siRNAs) (Vaucheret and Voinnet, 2024). However, this classification became more complex with new data from RNA sequencing experiments, leading to the identification of additional sRNA subclasses. These include secondary small interfering RNAs (siRNAs), trans-acting siRNAs (tasiRNAs), phased siRNAs (phasiRNAs), siRNAs derived from endogenous inverted repeats (endoIR-siRNAs), natural antisense siRNAs (nat-siRNAs), heterochromatic siRNA (hc-siRNA), and RNA quality control siRNA (rqc-siRNA) (Lee and Carroll, 2018; Zhan and Meyers, 2023; Vaucheret and Voinnet, 2024). However, it is important to recognize that this classification can be misleading, as different pathways often share substrates and factors involved in small RNA biogenesis and function, blurring the lines between them. This interconnectedness underscores the complexity and sophistication of sRNA-mediated regulation in plants.

**Table 1:** Small RNA classes in plants.

Nomenclature	Full name	Origin	Biogenesis factors
miRNA	Micro RNA	*MIR* loci	Pol II, HYL1, DCL1, SE, HEN1 (Dolata et al., 2018; Zhan and Meyers, 2023)
tasiRNA	Trans-acting siRNA	*TAS* loci	miRNA, AGO1/7, RDR6, DCL4 (Zhan and Meyers, 2023; Vaucheret and Voinnet, 2024)
phasiRNA	Phased siRNA	*PHAS* loci	Pol II, miRNA, AGO1, RDR6, DCL4/5 (Zhan and Meyers, 2023; Vaucheret and Voinnet, 2024)
endoIR-siRNA	Endogenous inverted repeat-derived siRNA	Endogenous inverted repeats	DCL1/2/3/4 (Henderson et al., 2006; Dunoyer et al., 2007; Vaucheret and Voinnet, 2024)
nat-siRNA	Natural antisense siRNA	Overlapping loci	DCL2/3/4 (Zhan and Meyers, 2023; Vaucheret and Voinnet, 2024)
hc-siRNA	Heterochromatic siRNA	Transposons	Pol IV, RDR2, DCL3, HEN1 (Matzke and Mosher, 2014; Cuerda-Gil and Slotkin, 2016; Erdmann and Picard, 2020; Zhan and Meyers, 2023; Vaucheret and Voinnet, 2024)
rqc-siRNA	Aberrant RNA	RNA quality control siRNA	RDR6, DCL4 (Martínez de Alba et al., 2015; Krzyszton and Kufel, 2022; Vaucheret and Voinnet, 2024)
easiRNA	Epigenetically activated siRNA	Activated transposons	Pol II, miRNA, AGO1, RDR6, DCL4 (Zhan and Meyers, 2023; Vaucheret and Voinnet, 2024)
vsiRNA	Virus-derived siRNAs	Viruses	RDR1/2/6, DCL2/3/4 (Baulcombe, 2022; Vaucheret and Voinnet, 2024)
risiRNA	Ribosomal siRNA	Pre-rRNA	RDR1/6, DCL2/4 (Lange et al., 2011; You et al., 2019; Hang et al., 2023)

Most miRNA precursors (pri-miRNAs) are transcribed by the polymerase II (Pol II) complex as capped and polyadenylated independent transcripts that fold to create hairpin structures with imperfect complementarity. They are processed by the microprocessor complex, composed of three core proteins: HYL1, DCL1, and SE, and their maturation is enhanced by multiple proteins (Dolata et al., 2018). Mature miRNAs bind to AGO proteins (AGO1, 2, 7, 10), forming RNA silencing complexes that target complementary mRNAs or non-coding RNAs (ncRNAs). This triggers cleavage of target RNAs or leads to translational repression followed by RNA decay (Zhan and Meyers, 2023; Vaucheret and Voinnet, 2024).

The canonical siRNA biogenesis pathways have been described previously in detail (de Felippes and Waterhouse, 2020; Vaucheret and Voinnet, 2024). They involve processing long, perfectly paired dsRNAs by the endonucleases DCL4 or DCL2. The resulting short 21–22 bp duplex siRNAs are then loaded onto AGO proteins, where one strand of the duplex is degraded, forming an RNA-induced silencing complex (RISC). The RISC utilizes the nucleotide sequence of the siRNA to identify and target cellular mRNAs for degradation, leading to gene silencing. The production of secondary siRNAs, which may arise from cleaved fragments, can further enhance this silencing effect. This process enables the amplification of siRNA production through a mechanism known as transitivity (de Felippes and Waterhouse, 2020; Sanan-Mishra et al., 2021; Tan et al., 2024). Also, some miRNA target mRNAs can serve as a source of secondary siRNAs. The cleaved RNA fragments have been shown to bind the AGO1 complex, which recruits one of the RNA-dependent RNA polymerases in Arabidopsis, RDR6. This enzyme creates dsRNA substrates, which are then processed by DCL2 and DCL4 (de Felippes and Waterhouse, 2020; Zhan and Meyers, 2023; Vaucheret and Voinnet, 2024). In specific cases, secondary siRNAs can originate from certain non-coding RNAs (ncRNAs), such as TAS precursors or retrotransposons, which generate epigenetically activated siRNAs (easiRNAs) (Zhan and Meyers, 2023; Vaucheret and Voinnet, 2024). A unique subclass of small RNAs that arises from transitivity and requires miRNA cleavage for their formation is termed phasiRNAs. This name reflects their generation mechanism, which involves multiple cleavages by DCL4 in a specific phased pattern relative to the primary miRNA binding site. The phasiRNA class also includes a particular group of tasiRNAs, which are produced from specific TAS precursors and target other transcripts *in trans* (Fei et al., 2013; Sanan-Mishra et al., 2021; Zhan and Meyers, 2023). One of the key factors in secondary siRNA biogenesis is the RNA-binding protein SGS3, which interacts with RDR6 (Fei et al., 2013; Elmayan et al., 2025). SGS3 also interacts with chromatin remodelers CHR11/17, which bind to transgene or endogenous loci that produce siRNAs. It has been proposed that SGS3 is recruited by CHR11/17 to these loci and shuttles between the nucleus and cytosol to facilitate RNA export and initiate siRNA production (Elmayan et al., 2025).

Plant genomes also produce long RNA hairpin structures with perfect or near-perfect self-complementarity that generate endoIR-siRNAs, also known as hp-siRNAs. Their synthesis depends, in part, on each of the DCL proteins (Henderson et al., 2006; Dunoyer et al., 2007; Vaucheret and Voinnet, 2024). Finally, the pairing of independently synthesized antisense transcripts can lead to the formation of nat-siRNAs, which have specific biogenesis factor requirements influenced by their loci (Zhan and Meyers, 2023; Vaucheret and Voinnet, 2024).

An important role of plant sRNA is to maintain genome integrity and stability, primarily at the transcriptional level. To combat the potential threat posed by harmful transposable elements, plants have developed a sophisticated and highly effective suppression system, namely RNA-directed DNA methylation (RdDM; **Figure 1**) (Matzke and Mosher, 2014; Cuerda-Gil and Slotkin, 2016; Erdmann and Picard, 2020; Zhan and Meyers, 2023). This mechanism utilizes plant-specific DNA-dependent RNA polymerases IV and V (Pol IV and Pol V) to silence detrimental genomic regions. Pol IV synthesizes short transcripts quickly converted into dsRNA by RNA-dependent RNA polymerase RDR2. These dsRNAs are then processed by the Dicer-like enzyme DCL3 into hc-siRNAs (also known as p4-siRNAs) (Zhan and Meyers, 2023; Vaucheret and Voinnet, 2024). These specialized sRNAs are incorporated into silencing complexes with AGO4, AGO6, and AGO9 proteins, which, along with DNA methyltransferases DRM1 and DRM2, specifically target transcripts produced by Pol V. Pol V transcripts act as scaffolds to guide silencing complexes to precise genomic locations. The hc-siRNAs provide sequence specificity for the transcriptional silencing mechanism, resulting in DNA methylation at targeted regions of the genome, particularly those densely populated with transposons and DNA repeats. This methylation recruits a variety of proteins responsible for maintaining TGS, including those involved in chromatin remodeling, histone modifications, preservation of DNA methylation, and stabilization of non-coding RNAs (Matzke and Mosher, 2014; Cuerda-Gil and Slotkin, 2016; Erdmann and Picard, 2020; Zhan and Meyers, 2023).

In addition to their essential role in regulating gene expression, sRNAs have retained robust anti-viral functions (Baulcombe, 2022; Vaucheret and Voinnet, 2024). During viral infections, virus-derived siRNAs (vsiRNAs) are generated from viral RNA through the action of endogenous RDR1 and RDR6 polymerases, along with DCL4 and, to a lesser extent, DCL2. The vsiRNAs are then bound by AGO1 and AGO2, which slice the viral RNA, creating an effective defense mechanism for the plant cell (Baulcombe, 2022; Vaucheret and Voinnet, 2024). Additionally, DNA viruses have been observed to trigger a silencing response akin to TGS, involving DCL3 and AGO4 (Baulcombe, 2022; Vaucheret and Voinnet, 2024). Remarkably, it appears that most factors associated with TGS and PTGS are capable of conferring immunity against various types of viruses, prompting these pathogens to evolve an array of anti-RNAi strategies (Pumplin and Voinnet, 2013; Baulcombe, 2022).

## RNA silencing triggers

Both dsRNA and single-stranded RNA (ssRNA) can trigger RNAi pathways; however, ssRNA requires the generation of dsRNA through the activity of one of the RDRs. Under normal conditions, these enzymes target only a limited number of dedicated endogenous transcripts. This indicates the presence of specific recruitment mechanisms for RDR polymerases or proteins that protect transcripts from dsRNA production. Initial insights into this process came from analyzing transgene silencing in Arabidopsis (Stam et al., 1997). Silencing of transgenes requires components of sRNA pathways, including RDR6, DCL2/4, and AGO1, and may lead to decreased expression of homologous sequences in the genome in a process called cosuppression (Stam et al., 1997). Only a subset of transformed lines typically exhibit repressed expression, raising the question of what signals trigger silencing. Several studies suggest that the number of transgene copies and the strength of transgene transcription are the primary causes of silencing (Stam et al., 1997; Lechtenberg et al., 2003; Schubert et al., 2004; Luo and Chen, 2007). It has been proposed that high transgene expression is associated with an increased misprocessing during transgene mRNA maturation, and the resulting aberrant transcripts attract RNAi machinery (de Felippes and Waterhouse, 2020; Krzyszton and Kufel, 2022). Aberrant RNAs that arise from errors in transcription or RNA maturation often lack 5' cap or poly(A) tail, or might contain premature termination codons, and are normally degraded by RNA quality control mechanisms (RQC), including nonsense-mediated decay (NMD) (Liu and Chen, 2016; Vaucheret and Voinnet, 2024). The hypothesis of aberrant RNA-triggered silencing was confirmed in numerous studies based on reporter transgenes and different mRNA maturation and degradation mutants, as described below.

## 5′ cap structure and RNA decapping are linked to siRNA production

**Figure 2:**
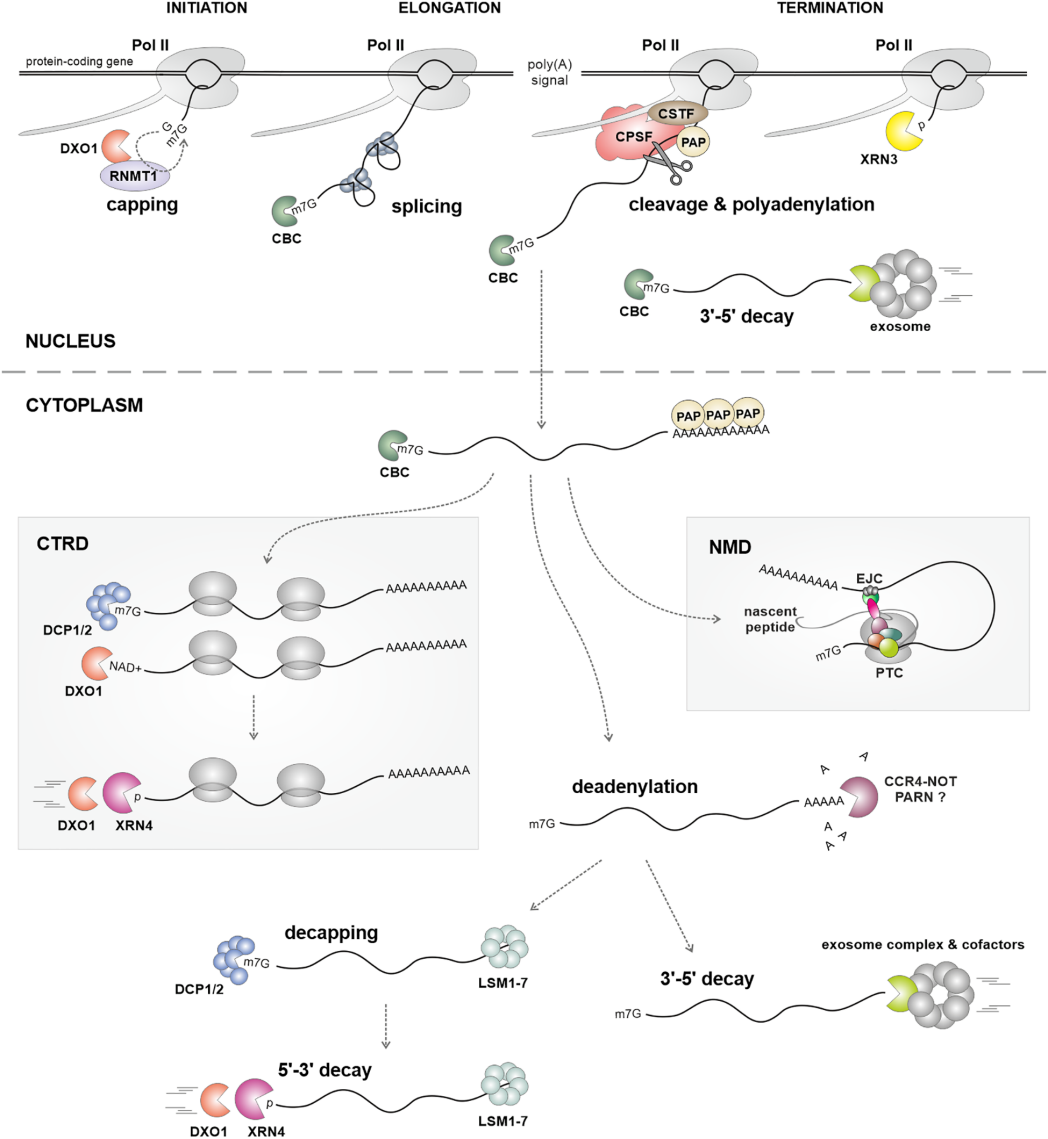
The main steps of RNA metabolism, from transcription to mRNA decay, with enzymes and factors involved in each step. Defects in these processes can act as sources of siRNAs. Enzymes and factors involved in each step are depicted in each panel. Known plant mutant lines in RNA metabolism factors that show defects in PTGS, either for transgenes or endogenous transcripts, are listed in **Table 2**. CTRD - co-translational mRNA decay; NMD - nonsense-mediated decay; PTC - premature termination codon.

The m^7^G cap protects the RNA 5' end from degradation and facilitates the recruitment of factors engaged in splicing, transcription elongation and termination, nuclear export, and translation (Gonatopoulos-Pournatzis and Cowling, 2014; Avila-Bonilla and Macias, 2024; Potužník and Cahova, 2024). This is possible through functions of the cap-binding complex (CBC), consisting of CBP20, ABH1 (CPB80), and SERRATE (SE) (Gregory et al., 2008; Laubinger et al., 2008; Raczynska et al., 2010; Li et al., 2016). The removal of the cap is essential for the degradation of mRNA and is carried out in the cytoplasm by the decapping complex (**Figure 2**), which consists of the catalytic subunit DCP2 and its cofactor DCP1, along with several other components such as DCP5, DHH1, VCS, the LSM1–7 complex, and PAT1 (Maldonado-Bonilla, 2014; He and Jacobson, 2023). Both decapping complexes and mRNAs can be found in distinct cytoplasmic structures known as processing bodies, or P-bodies (Maldonado-Bonilla, 2014; He and Jacobson, 2023; Kearly et al., 2024). Dysfunctional decapping in the Arabidopsis Col-0 ecotype causes strong developmental phenotypes leading to post-embryonic lethality, suggesting a pivotal role of 5'-3' mRNA degradation (Maldonado-Bonilla, 2014). However, enhanced degradation of mRNA from the 3' end, observed in other Arabidopsis ecotypes, can suppress these strong phenotypes (Zhang et al., 2010). Interestingly, lethality but not sterility of *dcp2–1* and *vcs-6* mutants can be suppressed by a mutation in the *RDR6* gene (Martínez de Alba et al., 2015). Both decapping mutants accumulate small RNAs, mainly 21 nucleotides in length, generated from hundreds of mRNAs, and partially dependent on the RDR6 activity (**Table 2**) (Martínez de Alba et al., 2015). Since these siRNAs are produced only in plants with defects in RNA degradation pathways, they are referred to as RNA quality control siRNAs (rqc-siRNAs) (Martínez de Alba et al., 2015; Krzyszton and Kufel, 2022; Vaucheret and Voinnet, 2024). Moreover, *dcp1*, *dcp2*, and *vcs* mutations enhance transgene PTGS (**Table 2**) (Thran et al., 2012; Martínez de Alba et al., 2015), which, at least in the case of *dcp2*, is also dependent on RDR6 and associated with a decrease in the level of uncapped mRNA (Thran et al., 2012). In contrast, the lack of the decapping activator LSM1 causes only limited accumulation of rqc-siRNAs, suggesting that only mutations with a strong impact on RNA decay can induce the production of rqc-siRNAs (Krzyszton and Kufel, 2022).

**Table 2:** Mutations in RNA metabolism genes that affect RNAi.

Protein/ complex	Function	Transgene silencing	Endogenous siRNA production
DXO1	cap methylation, CTRD	–	*dxo1-2* (Kwasnik et al., 2019; Pan et al., 2020)
Spliceosome & cofactors	splicing	*esp3-1* (Herr et al., 2006) *smd1b* (Elvira-Matelot et al., 2016)	–
CPA	cleavage & polyadenylation	*esp1-1* (*CSTF64*) *esp4-1, esp4-3* (*Symplekin*) *esp5-1* (*CPSF64*) (Herr et al., 2006)	*cstf64-2* (Krzyszton and Kufel, 2022)
XRN3	transcription termination	*xrn3-3* (Gy et al., 2007)	*xrn3-8* (Krzyszton et al., 2018)
Decapping complex & activators	decapping	*its1* (*DCP2*) (Thran et al., 2012) *dcp1-3, vcs-6, vcs-8, vcs-9* (Martínez de Alba et al., 2015)	*dcp2-1*, *vcs-6* (Martínez de Alba et al., 2015) *lsm1a lsm1b* (Krzyszton and Kufel, 2022)
XRN4	5′-3′ mRNA decay, CTRD	*xrn4-1* (Gazzani et al., 2004; Gy et al., 2007) *xrn4-5* (Parent et al., 2015b; Yu et al., 2015)	*ein5-6* (Gregory et al., 2008) *ein5–1 ski2-3* (Zhang et al., 2015)
Exosome complex & cofactors	3′-5′ mRNA decay	*rrp4^iRNAi^*, *rrp41^iRNAi^*, *amiR-RRP44A, rrp6l1* (Moreno et al., 2013) *ski2-4* (Branscheid et al., 2015) *ski3-3* (Yu et al., 2015) *hen2-1* (Lange et al., 2014) *sop1-5* (Hématy et al., 2016)	*cer7-3* (*RRP45B*), *ski2-6*, *ski3-7*, *ski8-7* (Zhao and Kunst, 2016) *ski2-4* (Branscheid et al., 2015) *ein5–1 ski2-3* (Zhang et al., 2015) *atrimmer1/rrp6l1* (Ye et al., 2016) *ski2-5, ski3-5, cer7-3* (*RRP45B*)*, rrp4-2, hen2-5* (Vigh et al., 2022)
CCR4-NOT/ PARN	deadenylation	*ccr4a*, *ahg2-1* (*PARN*) (Moreno et al., 2013)	–
NMD factors	NMD	*upf1-6*, *upf3-3* (Moreno et al., 2013)	*upf1-5*, *upf3-1* (Krzyszton and Kufel, 2022)

A notable interaction between cap turnover and RDR6-dependent small RNA production was observed in mutants of the DXO1 protein (**Figure 2**; **Table 2**). This enzyme plays a role in the biogenesis of mRNA 5′ cap by promoting m^7^G cap methylation by the RNMT1 methyltransferase, and possibly also in the mRNA 5′ end quality control by eliminating the noncanonical NAD^+^ cap (a process known as deNADding) (Kwasnik et al., 2019; Pan et al., 2020; Yu et al., 2021; Xiao et al., 2023; Zakrzewska-Placzek et al., 2025). In addition, it contributes to the cytoplasmic degradation of ribosome-associated mRNAs via the cotranslational mRNA decay (CTRD) mechanism (Carpentier et al., 2025; Deragon and Merret, 2025). Our research using *dxo1* mutants revealed a significant accumulation of rqc-siRNAs, primarily generated from mRNAs that typically do not produce siRNAs (Kwasnik et al., 2019). Significantly, the accumulation of rqc-siRNAs was inhibited in the *dxo1/rdr6* double mutant (Kwasnik et al., 2019).

The decapping-mediated removal of mRNAs is thought to protect these molecules from being converted into small RNAs, which could negatively impact gene expression. This mechanism seems highly effective, as it is utilized by plant DNA viruses, specifically geminiviruses, to boost their proliferation (Ye et al., 2015). One of the viral proteins, BV1, can induce the expression and nuclear export of ASYMMETRIC LEAVES 2 (AS2), which serves as an endogenous enhancer of DCP2 enzymatic activity in P-bodies (Ye et al., 2015). Plants that overexpress AS2 exhibit increased susceptibility to infection, while the *as2* mutant demonstrates greater resistance. Additionally, when AS2 is overexpressed, mRNAs from silenced reporter transgenes are upregulated, and the corresponding siRNAs decrease (Ye et al., 2015). This indicates that the siRNA pathway is significantly more effective at inhibiting virus replication than RNA degradation. If the balance is tipped toward RNA decay, cells become more vulnerable to infection (Ye et al., 2015). However, the effect may be virus-type-specific as a *dcp2* mutation leads to increased accumulation of the Turnip rosette virus (TRV) ssRNA while also enhancing virus-induced gene silencing (VIGS) (Ma et al., 2015). Nevertheless, P-bodies and siRNA bodies, which contain RDR6 and SGS3, are often found in close proximity in the cytoplasm, highlighting the connection between RNA decapping and siRNA production (Martínez de Alba et al., 2015).

## The contribution of 5′-3′ exoribonucleases to siRNA-mediated regulation

In addition to mRNA decapping, the production of rqc-siRNA from endogenous mRNAs significantly increases when both the 5′-3′ and 3′-5′ cytoplasmic RNA degradation pathways are not functioning correctly. If either of these mechanisms is impaired alone, the accumulation of sRNAs increases, but this occurs only for a limited number of loci or reporter transgenes (Gregory et al., 2008; Zhang et al., 2010, Zhang et al., 2015; Shin et al., 2013; Branscheid et al., 2015; Hématy et al., 2016; Krzyszton and Kufel, 2022).

The key enzymes responsible for 5'-3' RNA degradation in Arabidopsis are XRN2-4, representing a conserved family of 5'-3' XRN exoribonucleases. XRN2 and XRN3 are primarily localized in the nucleolus and nucleus, respectively, and have overlapping roles in rRNA maturation. However, XRN2 is more critical for this process, while XRN3 also plays a key role in Pol II transcription termination (Zakrzewska-Placzek et al., 2010; Nagarajan et al., 2013; Kurihara, 2017; Krzyszton et al., 2018). In contrast, the cytoplasmic XRN4 protein participates in the general degradation pathway of decapped mRNAs and, alongside DXO1, in the CTRD mechanism (**Figure 2**) (Merret et al., 2013; Maldonado-Bonilla, 2014; Yu et al., 2016; Carpentier et al., 2020, Carpentier et al., 2024). Additionally, XRN4 degrades mRNA 3' cleavage products generated by miRNAs and contributes to the removal of mRNAs targeted by a specific class of nat-siRNAs known as long siRNAs (Nagarajan et al., 2013). All Arabidopsis XRN proteins, as well as DXO1, are inhibited by adenosine 3′,5′-diphosphate (PAP), which is increased in mutants of the *FRY1* gene encoding nucleotidase responsible for PAP hydrolysis in plants (Gy et al., 2007; Chen et al., 2011; Kwasnik et al., 2019).

**Figure 3:**
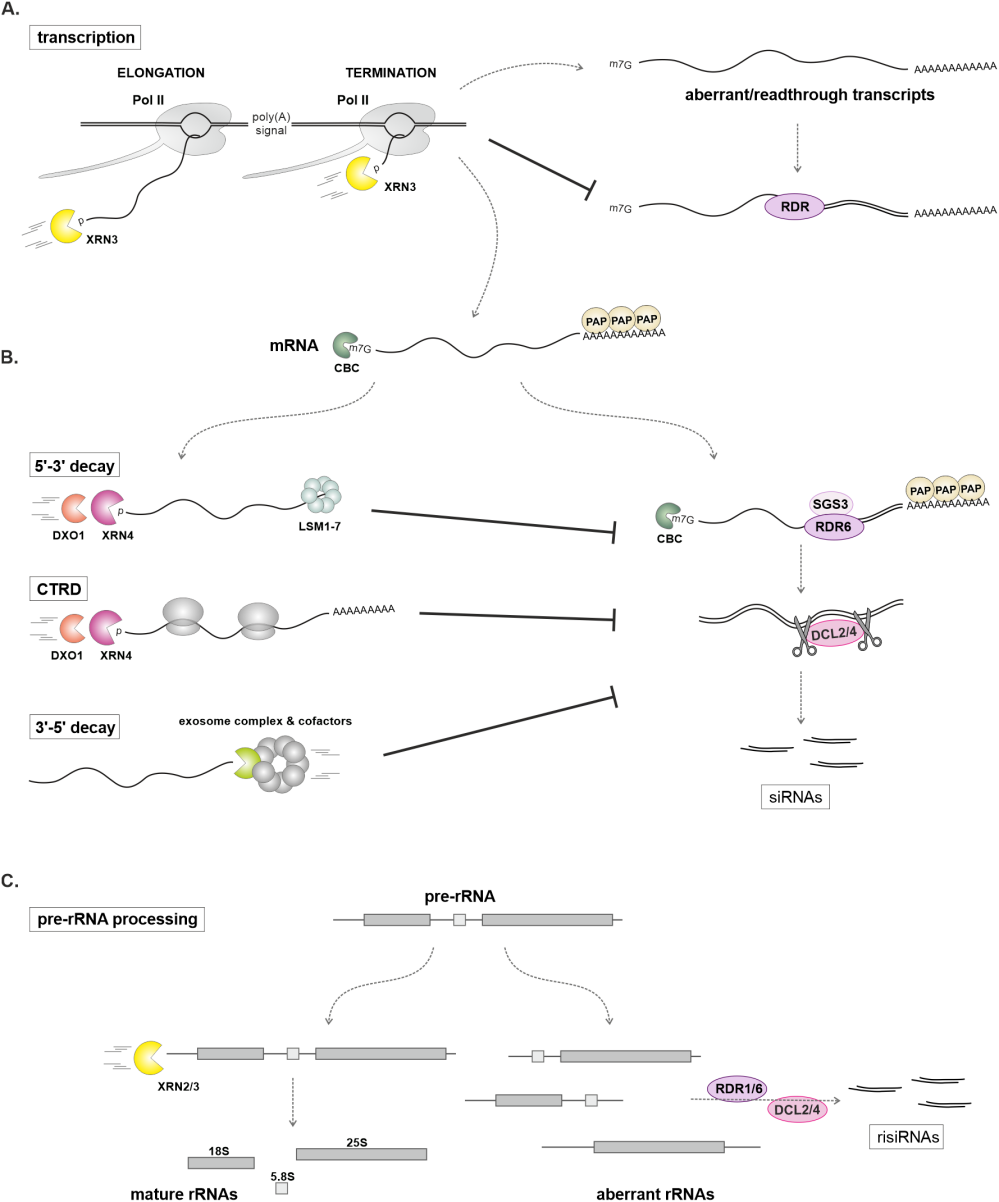
The role of exoribonucleases as PTGS suppressors. **(A)** In the nucleus, the 5′-3′ exoribonuclease XRN3 degrades nascent transcripts during polymerase II (Pol II) transcription elongation and participates in termination. This prevents the synthesis of aberrant or readthrough transcripts that could serve as substrates for RNA-dependent RNA polymerase (RDR). **(B)** In the cytoplasm, both 5′-3′ and 3′-5′ mRNA-degrading enzymes efficiently remove aberrant or superfluous mRNAs that could otherwise become substrates for siRNA generation via the SGS3-RDR6-DCL2/4-dependent pathway. In the absence of exoribonucleases, increased levels of siRNAs are observed in Arabidopsis mutants (**Table 2**). **(C)** The exoribonucleases XRN2 and XRN3 are involved in the processing of ribosomal RNA precursors (pre-rRNA). Defects in this process lead to the production of ribosomal siRNAs (risiRNAs), which depend on the DCL2/4 and RDR1 (Hang et al., 2023) or RDR6 (You et al., 2019).

A disturbance in mRNA 5′-3′ degradation significantly impacts the accumulation of siRNAs (**Figure 3**). A mutation in the *XRN4* gene leads to increased silencing of transgenes (**Table 2**), an effect that can be suppressed by a mutation in *RDR6* (Gazzani et al., 2004). Interestingly, the role of XRN4 in transgene silencing suppression may be organ-specific (Vogel et al., 2011), and enhanced silencing in the *xrn4* mutant can lead to co-suppression (Hayashi et al., 2012). Defective transgene silencing in the *ago1* mutant can be restored by *xrn4* or *fry1* mutations, confirming the role of cytoplasmic 5′-3′ RNA degradation as a mechanism that limits PTGS (Gy et al., 2007; Yu et al., 2015). Consistent with the enzymatic function of XRN4, decapped transgene mRNA accumulates in the *xrn4 rdr6* double mutant (Gazzani et al., 2004; Gy et al., 2007; Yu et al., 2015; Zhang et al., 2015). A similar phenomenon occurs with endogenous mRNAs; in *xrn4* plants, more than a hundred accumulated uncapped transcripts are a source of 21 nt siRNAs produced from both strands (**Table 2**) (Gregory et al., 2008). The extent of accumulation of these sRNAs may depend on the involvement of XRN4 in the CTRD, which may influence siRNA biogenesis (Gregory et al., 2008; Wroblewski et al., 2014; Yu et al., 2016).

In the *xrn4* mutant, the accumulation of 21 nt siRNAs from both mRNA strands is greatly increased when cytoplasmic 3'-5' mRNA degradation is additionally disrupted by a hypomorphic mutation in the *SKI2* gene, which encodes a component of the exosome-associated SKI complex (Zhang et al., 2015) (see below). The *xrn4 ski2* double mutant plants produce large amounts of siRNAs from hundreds of protein-coding genes and show genome-wide changes in mRNA levels. Importantly, the full double knockout of XRN4 and SKI2 results in lethality (Zhang et al., 2015), but all phenotypes observed in the *xrn4 ski2* plants are rescued by mutations in the PTGS pathway, including *rdr6*, *ago1*, *sgs3*, and double *dcl2 dcl4* mutants. This suggests that *xrn4 ski2* lethality stems from the production of unwanted siRNAs (Zhang et al., 2015).

Both nuclear XRN2 and XRN3 also act as endogenous transgene silencing suppressors, although to a lesser extent than XRN4, potentially acting in an organ-specific manner (Gy et al., 2007; Vogel et al., 2011; Elvira-Matelot et al., 2016). Consistent with the known functions of these nucleases, mutations in *XRN2* and *XRN3* genes, along with *FRY1*, result in the production of ribosomal siRNAs (risiRNAs) from pre-rRNA fragments that accumulate in these plants (Lange et al., 2011; You et al., 2019). Interestingly, risiRNAs bind to AGO1 and AGO2 proteins, competing with miRNAs that normally form complexes with these proteins, ultimately reducing miRNA abundance (You et al., 2019). In turn, the XRN3 enzyme contributes to the Pol II termination mechanism, which is crucial for limiting the undesirable production of siRNAs ( (Krzyszton et al., 2018); see the section on the role of transcription termination in RNA silencing).

The role of XRN proteins as PTGS suppressors represents an important mechanism by which the elimination of decapped mRNA can prevent unwanted gene silencing through facilitating rapid degradation. This may be particularly true for highly expressed genes that are more susceptible to aberrant or inefficient mRNA processing (Zhang et al., 2015).

## 5′-3′ RNA degradation machinery as a suppressor of gene silencing

RNA degradation and the processing of various classes of transcripts from the 3' end are performed by the exosome complex (**Figures 2**, **3**) (Lange and Gagliardi, 2022). In Arabidopsis, the core of this complex consists of nine proteins: RRP40-43, RPP45A/B-46, RRP4, MTR3, and CSL4. While the RRP41 subunit may exhibit phosphorolytic enzymatic activity, most exosome-mediated processes are carried out by its associated cofactors. These include the 3'-5' exoribonucleases RRP44A/B and RRP6L1-3, along with helicases and RNA-binding proteins (Lange and Gagliardi, 2022). These cofactors play a crucial role in determining the exosome substrate specificity in different cellular compartments. In the nucleolus, the exosome-mediated activities are supported by RRP44A, RRP6L2, and helicase MTR4, which are involved in rRNA processing and the removal of excess pre-rRNA fragments (Lange et al., 2011; Kumakura et al., 2013). In the nucleoplasm, SOP1 and helicase HEN2 support the degradation of diverse polyadenylated RNAs, including intergenic, pseudogenes, improperly spliced mRNAs, snoRNAs, and miRNA precursors (Lange et al., 2014; Hématy et al., 2016). Finally, in the cytoplasm, RRP44B (SOV) and the SKI2/3/7/8 complex contribute to mRNA decay (Zhang et al., 2010, Zhang et al., 2015; Kumakura et al., 2013), whereas RST1 and RIPR proteins participate in RNA quality control and prevent the unwanted silencing of endogenous genes (Lange et al., 2019; Auth et al., 2021). Nevertheless, some cofactors may function independently of the core exosome. For instance, nuclear RRP6L1 plays a role during TGS by stabilizing Pol V and enhancing the retention of Pol V-transcribed noncoding RNAs on chromatin (Zhang et al., 2014).

Loss-of-function mutations in genes encoding most of the exosome core components and *RRP44A* are lethal, which makes inferring their molecular role problematic. Analysis of knockdown mutant lines obtained using RNA silencing approaches, namely *RRP4^iRNAi^*, *RRP41^iRNAi^*, and *amiRNA-RRP44A* mutant lines, revealed enhanced transgene PTGS, mainly mediated by 21-nucleotide siRNAs derived from the dsRNA produced by RDR6 and SGS3 (Moreno et al., 2013). However, high-throughput sequencing of small RNAs from *RRP4^iRNAi^* and *RRP41^iRNAi^* lines showed that the knockdown of these core subunits had little effect on siRNA production from endogenous sources (Shin et al., 2013; Hématy et al., 2016).

The alternative exosome subunit CER7 (RRP45b) protects some endogenous mRNAs in the cytoplasm from the production of unwanted siRNAs (Lam et al., 2015; Vigh et al., 2022). A mutation in *CER7* leads to the accumulation of siRNAs from the *CER3* gene encoding a cuticular wax biosynthetic enzyme and at least five other protein-coding genes, resulting in mRNA downregulation, defects in wax deposition and glossy stem phenotype (Hooker et al., 2007; Lam et al., 2012). This phenotype was also observed in plants lacking exosome cofactors RST1 and RIPR (Lange et al., 2019; Yang et al., 2020). The effects of *cer7* mutation can be suppressed by mutations in *AGO1*, *SGS3*, *HEN1*, and both *RDR1* and *RDR6*, showing that the wax-deficient phenotype is caused by *CER3* mRNA silencing (Lam et al., 2012, Lam et al., 2015). Furthermore, a weak *dcl4* mutant also ameliorates the defective wax deposition, although knockouts of *DCL4* or its cofactor *DRB4* in a *cer7* background are lethal (Lam et al., 2015). Surprisingly, mutations in *SKI2*, *SKI3*, or *SKI8* also suppress the *cer7* phenotype and reduce siRNA production from *CER3* mRNA, even though the SKI complex is an exosome cofactor (Zhao and Kunst, 2016).

The cytoplasmic SKI complex plays a crucial role in degrading the 5' cleavage fragments of miRNA targets (Branscheid et al., 2015; Vigh et al., 2022). When this process is deficient, it results in the production of low-abundance, mostly RDR6-dependent 21 nt siRNAs originating from regions near the cleavage site. While the majority of siRNAs arise from the 5' cleavage fragments stabilized in the *ski2* mutant, some are also produced from non-accumulating 3' fragments (Branscheid et al., 2015). Interestingly, the direction of siRNA transitivity can be anticipated based on the asymmetry in the strength of pairing between the miRNA and its target. This suggests that the role of the SKI complex in siRNA production is not solely dependent on the degradation of miRNA cleavage fragments, and it may also involve the removal of the AGO1 complex prior to the recruitment of RDR6 (Branscheid et al., 2015). Supporting the role of SKI2 in miRNA-triggered transitivity, among fewer than 200 mRNAs with increased levels of siRNAs in the *ski2* mutant, 20% are identified as miRNA targets. The number of siRNA-producing genes is significantly elevated in the double *xrn4 ski2* line, as described above (Zhang et al., 2015). In a context unrelated to miRNA cleavage, a *ski2* mutation enhances RDR6-dependent PTGS of transgenes (Zhang et al., 2015). Similarly, *ski3* has been shown to restore transgene silencing that is de-repressed in the *ago1* mutant (Yu et al., 2015). Importantly, a direct comparison of *xrn4* and *ski3* mutants indicates that cytoplasmic RNA degradation from the 5' end contributes more significantly to the suppression of transgene silencing than degradation from the 3' end (Yu et al., 2015).

In eukaryotic cells, 3'-5' mRNA decay in the cytoplasm is initiated by the removal of the poly(A) tail by deadenylases, the CCR4-NOT and PAN2/3 complexes, along with PARN (Reverdatto et al., 2004; Liang et al., 2009; Arae et al., 2019; Armbruster et al., 2019). In flowering plants, however, homologues of PAN2/3 have not been identified (Pavlopoulou et al., 2013; Chantarachot and Bailey-Serres, 2018), and the role of PARN in cytoplasmic mRNA degradation is questionable due to its primarily mitochondrial localization (Hirayama et al., 2013; Kanazawa et al., 2020). Nevertheless, both *parn* and *ccr4a* mutants exhibit enhanced RDR6- and SGS3-dependent transgene silencing (**Table 2**) (Moreno et al., 2013), and the CCR4-NOT complex component NOT1 was identified in a genetic screen for RdDM regulators in *Arabidopsis* (Zhou et al., 2020). However, it was shown recently that CCR4a regulates a distinct set of transposable elements than those controlled by RDR6, acting independently of the siRNA pathway (Wang et al., 2024).

Nucleoplasmic exosome cofactors HEN2, SOP1, and RRP6L1 also act as endogenous suppressors of transgene PTGS (Moreno et al., 2013; Lange et al., 2014; Hématy et al., 2016). More importantly, RRP6L1 has a crucial role in the production of DCL-independent siRNAs from Pol II transcripts, which likely trigger TGS (Ye et al., 2016). In contrast, the nucleolar protein MTR4 contributes minimally to the suppression of transgene silencing due to its limited role in processing aberrant mRNAs (Lange et al., 2011, Lange et al., 2014).

Overall, the exosome and its cofactors appear to play a significant role in clearing aberrant mRNAs and protecting endogenous transcripts from PTGS. However, the phenotypic effects observed in mutants are weaker than anticipated. This may be attributed to the lethality associated with exosome knockouts or, as demonstrated by the *xrn4 ski2* double mutant, strong redundancies between the 5' and 3' decay pathways. These findings are further supported by observations that knockouts of RRP41 and RRP44A, as well as RRP44B, have no effect on the accumulation of viral RNA in plants (Kumakura et al., 2013).

## Crosstalk between nonsense-mediated decay and RNA silencing

Nonsense-mediated decay (NMD) is a cellular mechanism conserved in plants that safeguards against the translation of aberrant mRNAs containing premature stop codons (PTCs) (**Figure 2**). These PTCs often arise due to defective splicing or transcription errors, and if left uncontrolled, these aberrant transcripts can give rise to truncated protein products that not only lack functionality but may also be detrimental to cellular functions (Raxwal and Riha, 2023; Luha et al., 2024). However, the role of NMD extends beyond mere RNA quality control, as it has been demonstrated to play an important regulatory function in fine-tuning gene expression (Ohtani and Wachter, 2019; Raxwal and Riha, 2023; Luha et al., 2024). Many plant mRNAs display characteristics that render them susceptible to NMD, including upstream open reading frames (uORFs), long 3' untranslated regions (3′UTRs), and introns within the 3′UTR (Peccarelli and Kebaara, 2014).

While mutations in essential NMD factors like UPF1 and UPF3 result in the accumulation of NMD targets, these transcripts are not typically decapped or deadenylated. This may suggest that they may not be detected as aberrant by RNAi machinery. However, a number of studies revealed that *upf1* and *upf3* mutants enhanced RDR6- and SGS3-dependent transgene silencing. Moreover, UPF1 protein co-localizes with cytoplasmic siRNA-bodies associated with siRNA production (Moreno et al., 2013; Elvira-Matelot et al., 2016). It can be assumed that, in addition to PTCs, NMD substrates may possess other distinctive features of aberrant transcripts, such as stalled ribosomes that channel them into small RNA biogenesis pathways, as was shown for siRNA production from transposable elements (Kim et al., 2021). However, only a limited number of protein-coding genes showed increased siRNA production in *upf1* and *upf3* mutants, suggesting that specific features of NMD substrates may not be sufficient to induce siRNA biogenesis (Krzyszton and Kufel, 2022).

Surprisingly, NMD factors appear to limit the amplification of some plant RNA viruses by acting independently of small RNA pathways (Garcia et al., 2014; May et al., 2018; Chen et al., 2024), and some viruses, such as cucumber mosaic virus (CMV), have evolved mechanisms to evade NMD (Zhao et al., 2025). These findings indicate that the functions of NMD factors in PTGS may not be directly linked to RNA quality control.

## Defects in mRNA maturation provide substrates for siRNA production

**Figure 4:**
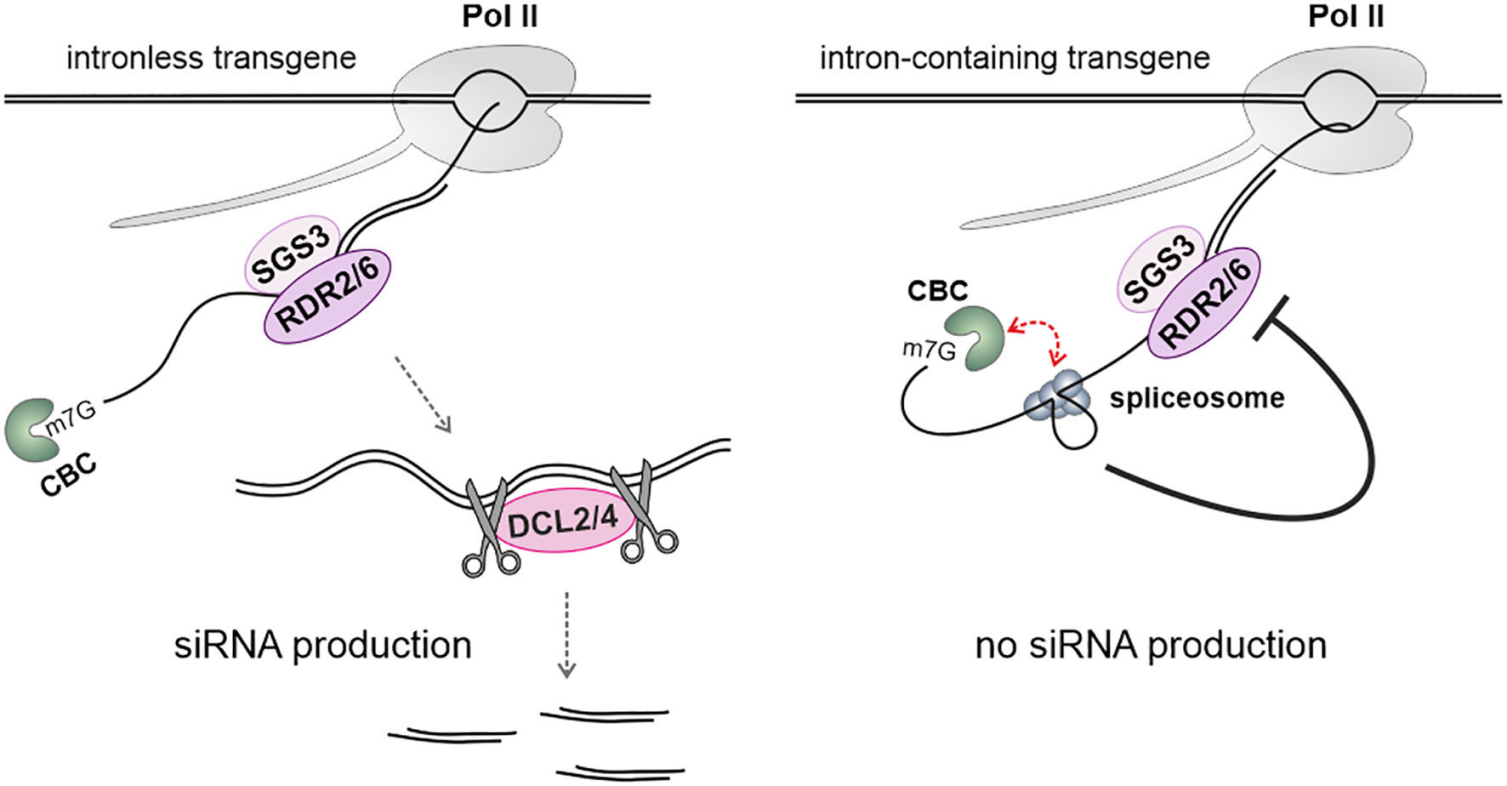
The effect of intron splicing on RNA silencing. Efficiently spliced introns attenuate the activity of the RNA-dependent RNA polymerases RDR6 and/or RDR2 along transcripts through a mechanism that requires the cap-binding protein ABH1, a component of the cap-binding complex (CBC). Pre-mRNA splicing entails interactions between the CBC and the spliceosome, adding structural complexity to the spliced transcript. This prevents the transcript from becoming a substrate for RDRs (Christie et al., 2011).

The processes of transcription elongation, mRNA processing, and transcription termination are error-prone, leading to the generation of abnormal mRNAs, with splicing errors being a primary source of these aberrations. Evidence from studies involving the yeast *Cryptococcus neoformans* shows that stalled spliceosomes can induce the production of siRNAs from mRNAs (Dumesic et al., 2013). In Arabidopsis, it has been observed that transgenes with spliced-out introns are less susceptible to silencing compared to those that are intronless or unspliced (Christie et al., 2011). This suggests that efficient splicing may help prevent transcripts from entering siRNA pathways (**Figure 4**). Additionally, spliced transgenes targeted by miRNAs undergo less efficient silencing than their intronless counterparts. The same also applies to endogenous mRNAs, as intronless genes are more prone to the production of small RNAs (Christie et al., 2011). Moreover, two proteins involved in splicing have been identified as endogenous suppressors of the PTGS in Arabidopsis: ESP3, a homolog of the yeast DEAH RNA helicase Prp2, and the core snRNP protein SmD1b (Herr et al., 2006; Elvira-Matelot et al., 2016). In the case of SmD1b, it binds to transcripts derived from silenced transgenes, but not from those that were not silenced. The presence of an intron in the transgene has a limited effect on the degree of suppression, and mutations in the *SMD1b* gene do not cause intron retention in the mRNA of silenced transgenes (Elvira-Matelot et al., 2016). The silencing defect observed in the *smd1b* mutant can be reversed by mutations in other genes that act as endogenous PTGS suppressors, such as *UPF3*, *XRN2*, *XRN3*, or *XRN4*. This indicates that SmD1b is not strictly essential for the silencing process. It has been suggested that SmD1b protects both intron-containing and intronless aberrant mRNAs from degradation in the nucleus, which allows for siRNA production in the cytoplasm (Elvira-Matelot et al., 2016).

Additionally, several splicing factors have been identified to play a role in TGS. The exact mechanism by which these factors influence TGS is not well understood, but it may involve interactions with the silencing machinery located in nuclear Cajal bodies (Ausin et al., 2012; Dou et al., 2013; Huang et al., 2013; Zhang et al., 2013; Du et al., 2015).

## The role of transcription termination in protecting genes from silencing

The maturation of mRNA 3' end involves cleavage of the nascent transcript followed by the addition of a poly(A) tail. This process is carried out by a multiprotein cleavage and polyadenylation complex (CPA) that is directed by specific terminator sequences in the pre-mRNA (**Figure 2**) (Shi and Manley, 2015). Studies using reporter transgenes have shown that defects in mRNA 3' end formation, caused by missing or ineffective terminator sequences, trigger the production of siRNAs and result in strong silencing effects. This phenomenon can lead to co-suppression and is dependent on RDR6 (Luo and Chen, 2007; Nicholson and Srivastava, 2009). In mutants lacking RDR6, transgenes that do not have proper terminator sequences generate non-polyadenylated read-through transcripts (Luo and Chen, 2007). These aberrant transcripts are believed to recruit RDR6, which in turn initiates silencing that can be suppressed by either XRN4 or SKI3 (Yu et al., 2015). Consequently, adding strong termination signals to transgenes significantly reduces their silencing (Luo and Chen, 2007; Nicholson and Srivastava, 2009; de Felippes and Waterhouse, 2020). In line with this, mutations in three putative components of the Arabidopsis cleavage and polyadenylation complex, namely homologs of human Symplekin/Pta1, CPSF100, and CstF64, cause transgene termination defects and enhance RDR6-dependent silencing (Herr et al., 2006). Also, in the case of endogenous mRNAs, there is a substantial accumulation of endogenous read-through transcripts in *cstf64* mutants, accompanied by an enrichment of small RNAs (**Table 2**) (Krzyszton and Kufel, 2022).

Once pre-mRNA is cleaved, Pol II continues transcription until it is caught up by XRN3, which degrades the nascent RNA. This degradation, called the “torpedo mechanism”, leads to the release of Pol II from the DNA template (**Figure 2**) (Kurihara, 2017; Krzyszton et al., 2018). If uncapped nascent RNAs are not efficiently removed after cleavage and polyadenylation, this may trigger the production of small RNAs from readthrough transcripts. It has been shown that higher levels of readthrough transcripts, which are antisense to the reporter transgene, result in stronger transgene silencing (Parent et al., 2015b). In turn, mutant lines, such as *xrn3* and *xrn4*, in which removal of these readthrough transcripts is impaired, have increased levels of siRNAs and enhanced PTGS (**Table 2**) (Parent et al., 2015b; Krzyszton et al., 2018). The subcellular localization of XRN4, which has a role in this process, suggests that some readthrough transcripts are exported to the cytoplasm. Low levels of uncapped readthrough transcripts can be converted into dsRNA, leading to the production of siRNAs that target both the aberrant transcript and the complementary antisense mRNA. Alternatively, readthrough transcripts can directly pair with mRNA to form dsRNA. In both scenarios, small RNAs can spread beyond the initial region of complementarity due to the generation of secondary siRNAs that enhance silencing. Consistent with this, biogenesis of sRNA is completely abolished in the *rdr6*, *sgs3*, and *ago1* mutants, as well as the *dcl2 dcl4* double mutant (Parent et al., 2015b).

## Aberrant RNAs as triggers of silencing

The comprehensive studies presented here have led to the development of a general model for the interaction between RNA turnover and small RNA pathways in Arabidopsis. The production of small RNAs from single-stranded RNAs, whether exogenous or endogenous, is initiated only when their degradation is significantly inhibited or their levels are exceptionally high (Zhang et al., 2015; de Felippes and Waterhouse, 2020; Krzyszton and Kufel, 2022). This is probably due to the primary role of PTGS in combating viral RNA (Pumplin and Voinnet, 2013; Li and Wang, 2019; Vaucheret and Voinnet, 2024). RNAi pathways can trigger the cascade of secondary siRNAs that enhance silencing (Sanan-Mishra et al., 2021; Vaucheret and Voinnet, 2024), allowing these siRNAs to effectively compete with rapid viral amplification. However, if this process accidentally targets endogenous transcripts, it can have deleterious consequences, such as silencing essential housekeeping mRNAs. Therefore, RNA degradation serves as the first line of defense against aberrant transcripts.

Under normal circumstances, RNA quality control mechanisms remove defective low-level transcripts, thereby safeguarding against activation of RNAi pathways (Liu and Chen, 2016). In contrast, high levels of viral transcription generate numerous misprocessed RNAs that can evade degradation. As a result, some of these misprocessed RNAs can be detected and neutralized by the sRNA-mediated antiviral defense mechanism. However, this poses risks to the cell; for example, small RNAs derived from exogenous sequences might inadvertently target endogenous mRNAs (Pumplin and Voinnet, 2013). Moreover, the activation of small RNA pathways to defend against invading viruses could disrupt their normal regulatory functions and lead to the production of novel siRNAs from both exogenous and endogenous substrates. In fact, viral infections have been shown to trigger the production of 21-nucleotide virus-activated small interfering RNAs (vasiRNAs) from various endogenous mRNAs (Cao et al., 2014; Liu and Chen, 2016; Vaucheret and Voinnet, 2024). These vasiRNAs are involved in regulating the expression of plant genes associated with virus resistance and pathogenicity (Cao et al., 2014; Guo et al., 2017, Guo et al., 2018). Notably, in the case of the *xrn4* mutant, which shows increased resistance to viruses, vasiRNAs accumulate at higher levels (Cao et al., 2014). This suggests that while the activation of RNA interference pathways facilitates the production of virus-derived siRNAs, it may also lead to the generation of siRNAs from endogenous transcripts.

The potentially harmful effects of viral infection can be mitigated by the virus-induced endoribonuclease RTL1, which removes double-stranded RNA substrates of Dicer-like proteins, thereby inhibiting the production of siRNAs (Shamandi et al., 2015; Sehki et al., 2023). Additionally, the generation of secondary siRNAs may be limited due to competition between Dicer-like proteins DCL2 and DCL4 for dsRNA substrates (Parent et al., 2015a). The 22-nucleotide siRNAs produced by DCL2 and bound by AGO1 are known to initiate the synthesis of secondary siRNAs and enhance PTGS. In contrast, the 21-nucleotide siRNAs generated by DCL4 may inhibit the secondary siRNA cascade and reduce silencing efficiency (Parent et al., 2015a).

The nature of endogenous aberrant RNAs causing silencing remains an open question. The absence of one of the mRNA binding complexes, such as CBC, the exon junction complex, or poly(A)-binding proteins, may be a key factor in identifying aberrant transcripts. However, whereas single mutations that affect RNA degradation or quality control pathways are sufficient to induce transgene silencing (Liu and Chen, 2016), endogenous transcripts initiate siRNA production only when both 5' and 3' mRNA degradation is impaired or when decapping is defective (Martínez de Alba et al., 2015; Zhang et al., 2015; Krzyszton and Kufel, 2022). This requirement for severe impairment of RNA decay to trigger siRNA production demonstrates that aberrant mRNAs accumulating at lower levels are most likely rapidly eliminated through overlapping pathways.

## Functional implications of the interplay between RNA turnover and RNAi

Small RNAs play essential roles in various developmental processes, including embryonic development, leaf and flower formation, and tissue patterning (Li et al., 2017; Singh et al., 2018). siRNAs and miRNAs are also integral to signaling pathways that regulate gene expression under stress conditions, making RNAi an essential mechanism for plant stress responses (Li et al., 2017; Brant and Budak, 2018; Luo et al., 2024; Xu et al., 2024). As discussed in this review, regulation by small RNAs involves multiple RNA metabolic pathways that are essential for both the biogenesis of small RNAs and their function as regulators of gene expression (**Table 2**; **Figure 3**). The mechanisms involved in RNA decay and processing play a crucial role in gene silencing and can either activate or inhibit RNAi in response to changes in the environment. Furthermore, the RNA turnover machinery can quickly remove stress-responsive transcripts or selectively stabilize certain mRNAs.

The relationship between RNAi and RNA turnover plays a crucial role in the mechanism of stress memory. This phenomenon enables plants to retain a record of previous stress experiences, allowing for quicker and more robust responses in the future (Crisp et al., 2016; Xu et al., 2024). After an initial exposure to stress, stress memory modulates gene expression through epigenetic mechanisms, which include DNA methylation and chromatin remodeling. This process is influenced by RNA-mediated gene silencing, including PTGS and RdDM (Crisp et al., 2016; Song et al., 2019; Xu et al., 2024). High-throughput sequencing studies have shown that miRNAs participate in transgenerational adaptation to drought and heat stress. For instance, miR156 and miR824 are involved in integrating stress memory with plant development (Stief et al., 2014; Szaker et al., 2019; Xu et al., 2024).

Similarly, siRNA-guided epigenetic mechanisms also play a significant role in propagating stress memory. Heat stress triggers the expression of HSFA2, a heat stress transcription factor, which leads to the degradation of the RNA-binding protein SGS3 (Liu et al., 2019). This degradation inhibits the biosynthesis of tasiRNAs and activates the H3K27me3 demethylase REF6, which derepresses HSFA2. Together, HSFA2 and REF6 form a positive feedback loop that transmits long-term epigenetic memory of heat stress by promoting the transgenerational degradation of SGS3 (Liu et al., 2019). This transcriptional memory mechanism operates through the tasiRNA-targeted gene *HTT5*, which accelerates flowering and reduces disease resistance (Liu et al., 2019). Additionally, another HSFA2 target, a retrotransposon known as *ONSEN*, is activated in response to heat stress and is shown to be transposed to the next generation (Ito et al., 2011; Matsunaga et al., 2015). *ONSEN* contains heat-responsive elements that can be inserted into new genomic locations in the offspring of heat-stressed mutants with a defective RdDM pathway, demonstrating that stress adaptation in plants can also be achieved through the activation of TEs (Ito et al., 2011; Matsunaga et al., 2015; Hayashi et al., 2020; Niu et al., 2022; Nozawa et al., 2022; Nguyen et al., 2025).

The mechanisms of RNAi and RNA turnover are vital for maintaining both genome stability and integrity, as well as for the development of new gene functions. This dynamic interplay not only helps prevent the spread of mobile genetic elements, serving as a protective mechanism for the genome, but also highlights the role of TEs as more than just "selfish" elements. TEs actively contribute to plant stress responses, playing a crucial role in both immediate defense mechanisms and long-term adaptation to environmental challenges (Crisp et al., 2016; Ito et al., 2011; Matsunaga et al., 2015; Niu et al., 2022; Nguyen et al., 2025). Interestingly, RdDM-dependent methylation of TEs regulates parental genome dosage in Arabidopsis through a mechanism involving TE-derived easiRNAs (**Table 1**), which target transcriptionally active TEs for degradation to prevent transposition (Martinez et al., 2018). This mechanism is essential for forming viable seeds, and pollen-delivered easiRNAs are crucial for transmitting epigenetic information across generations (Martinez et al., 2018). With this perspective, the complex pathways of RNA regulation can be seen as contributing to the evolution of new gene functions by silencing or modifying the expression of existing genes, or even controlling genome dosage in plants.

## Conclusion and perspectives

Insights into the role and mechanisms of action of small RNAs illuminate the connections between RNA metabolic pathways and RNA interference. The production of sRNAs is closely tied to the efficiency of mRNA degradation, which serves as a frontline defense system that eliminates abnormal mRNAs. This process prevents the synthesis of dsRNAs from aberrant transcripts, which can subsequently act as substrates for Dicer-like enzymes. In plants, highly selective and coordinated mRNA decay pathways dictate which mRNAs are degraded and which are utilized to produce small RNAs. The complexity of these pathways, along with their interconnections and intricate regulatory mechanisms, makes their study particularly challenging. Consequently, some aspects and elements of this network remain elusive, and their unraveling necessitates further research. For instance, is there a specific hierarchy of abnormal features that guide RNAs towards small RNA production? Why do transgenic reporter systems seem to be more prone to producing rqc-siRNAs compared to endogenous transcripts? And how can we use this knowledge to enhance and innovate crop protection technologies?

In terms of plant physiology, recent studies have highlighted the significant role of small RNAs in regulating plant resistance to biotic stress, including infections caused by bacteria, viruses, and fungi. These findings have been summarized and discussed in numerous review articles (Rose et al., 2019; Niu et al., 2021; Qiao et al., 2021; Tang et al., 2021; Bilir et al., 2022; Jiang et al., 2023; Septiani et al., 2025). As a result of these discoveries, RNA interference pathways have been used to enhance plant protection against pathogens. These approaches have led to the development of sRNA-based technologies for crop disease management, such as RNAi mediated by artificial microRNAs (amiRNAs), synthetic trans-acting siRNAs (syn-tasiRNAs), host-induced gene silencing (HIGS), and spray-induced gene silencing (SIGS) (Niu et al., 2021; Tang et al., 2021; Bilir et al., 2022). Such innovative strategies aim to cultivate plants with stable disease resistance while also improving the relationship between plant resilience and crop yield. However, it remains an open question how overexpression of exogenous RNAi sources used for pest control affects endogenous RNA-mediated pathways. It is conceivable that, as in the case of virus infection, this will trigger the production of new small RNAs, altering the balance between RNAi pathways and RNA turnover. In consequence, this may lead to undesirable secondary effects on crop yield and fitness, especially upon challenging environmental conditions. Extensive long-term studies on the molecular mechanisms of RNA synthesis, processing, and degradation using model plants may contribute to crop enhancement and protection.
